# Efficacy and safety of cytoreductive surgery combined with hyperthermic intraperitoneal chemotherapy for epithelial ovarian cancer: a systematic review and updated meta-analysis

**DOI:** 10.1186/s43046-025-00286-y

**Published:** 2025-04-30

**Authors:** Ahmad Azhar Marzuqi, Vincent Enrico Anderson, Latifa Gustina Adilazuardini, Imke Maria Del Rosario Puling, Nyoman Deva Pramana Giri, Alfred Julius Petrarizky

**Affiliations:** 1 University of Brawijaya, Malang, Indonesia; 2 RSUD Dr. Saiful Anwar, Malang, Indonesia

**Keywords:** Epithelial ovarian cancer, Cytoreductive surgery (CRS), Hyperthermic intraperitoneal chemotherapy (HIPEC), Overall survival (OS), Progression-free survival (PFS), Cost-effectiveness

## Abstract

**Background:**

The high incidence of primary and recurrent ovarian cancer after surgery imposes a significant economic burden. Cytoreductive Surgery combined with Hyperthermic Intraperitoneal Chemotherapy (CRS + HIPEC) shows promise as a treatment for epithelial ovarian cancer (EOC). This study aims to evaluate CRS + HIPEC’s potential to improve survival outcomes, such as overall survival (OS) and progression-free survival (PFS) while reducing adverse events and enhancing cost-effectiveness.

**Method:**

A literature review was conducted using the PRISMA framework on databases including Scopus, ProQuest, and PubMed, with quality assessment through the Newcastle–Ottawa Scale (NOS) and Risk of Bias (RoB) 2.0. Quantitative analysis employed RevMan 5.4.1 with a pooled randomized effect model using log [hazard ratio].

**Result:**

From 15 studies involving 1982 participants, OS analysis showed significantly higher survival in the CRS + HIPEC group (HR = 0.67, *p* < 0.0004). Although PFS was higher in this group, the result was not statistically significant (HR = 0.86, *p* = 0.46). Adverse events were more likely in the intervention group compared to control group (OR = 1.81, *p* < 0.0001). Cost analysis revealed that the Incremental Cost-effectiveness Ratio per Quality-Adjusted Life Year (ICER/QALY) remains below Indonesia’s GDP threshold.

**Conclusion:**

CRS + HIPEC shows potential benefits in EOC management, particularly in OS and PFS improvement, alongside manageable adverse events and favorable cost-effectiveness. However, study design heterogeneity, differences in HIPEC protocols, and variations in patient populations limit the generalization of outcomes. The difference in response to HIPEC between primary and recurrent EOCs still needs further explanation.

## Introduction

Epidemiological data shows that ovarian cancer is the 8 th most common cancer type worldwide and 3rd in Indonesian women [1]. According to the Global Cancer Incidence, Mortality and Prevalence (Globocan) data, there were 313,959 new cases and 207,252 deaths from ovarian cancer in 2020 [2]. This makes ovarian cancer one of the diseases with the biggest spending on handling by the government. Badan Penyelenggara Jaminan Sosial (BPJS), which is Indonesia’s social security institution, showed data in 2018 that ovarian cancer ranked third with an average total direct treatment cost of 168 million [3]. Other than financial problems, ovarian cancer patients are also burdened by the danger of other diseases that will befall them and a small life expectancy [4]. The study by Wulandari (2018) reinforces this by finding that anxiety in ovarian cancer patients is in line with the previous statement [4]. The incidence of recurrence of ovarian cancer also adds to the problem of costs in ovarian cancer patients [4].

Ovarian cancer is a disease where the epithelium is eroded due to physical trauma during ovulation, which can cause cumulative genetic alterations in the cells which the most common type is epithelial ovarian cancer (EOC) [5]. EOC is a result of cellular DNA damage that facilitates invagination in the cortical stroma, which will eventually be trapped and form cysts in the stroma that stimulate proliferation to form cancer cells [5]. Current efforts in treating EOC are diverse, including debulking surgery, neoadjuvant chemotherapy (NACT), cytoreductive surgery (CRS)*,* primary chemotherapy with neoadjuvant therapy, immunotherapy, vaccines, and radiation [6]. Previous studies using the method of CRS followed by chemotherapy showed positive results on the improvement of EOC patients with more prolonged survival and reasonable economic burden [7]. However, cost-effectiveness analyses conducted in Korea, the Netherlands, and the USA show that these interventions are still quite expensive, with low rates of recurrence, progression-free survival from solid tumors, and poor overall survival [8–10]. Systemic chemotherapy often has limited effectiveness in treating recurrent cancer in the stomach because the liver partially digests chemotherapeutic agents before they can reach the cancer cells [11]. As a result, the dose of chemotherapeutic agent delivered to the cancer cells is significantly reduced and effectiveness is lost. Increasing the dose of chemotherapy agents will cause more negative adverse events (AEs) in patients, making it difficult for them to complete the treatment [11].

The latest treatment that can be tried is hyperthermic intraperitoneal chemotherapy (HIPEC). HIPEC treatment overcomes this limitation by delivering high doses of chemotherapeutic agents directly to cancer cells in the abdomen [12]. HIPEC has been used in several countries, one of them being Malaysia, in the treatment of colorectal cancer with a high incidence rate [12]. HIPEC is usually performed as the last step in tumor CRS, with the duration of the HIPEC procedure being 90 to 110 min [12]. The chemotherapy is only in the stomach for a short period, and few substances are absorbed by the whole body, thus avoiding some of the AEs of conventional chemotherapy. Furthermore, this method allows cancer cells to be directly exposed to chemotherapeutic agents/substances, thereby increasing the effectiveness of treatment. Heating the chemotherapeutic agent/substance to an average temperature of 40 °C increases the permeability of cancer cells to the agent/substance, which allows cancer cells to be removed more effectively by the agent/substance [12].

Yet, the role of HIPEC differs between primary and recurrent EOC, reflecting variations in disease biology, treatment context, and evidence base. In primary EOC, HIPEC is commonly administered during Interval Debulking Surgery (IDS) following neoadjuvant therapy (either primary or interval), with the objective of eradicating residual microscopic disease. Conversely, in recurrent EOC, the application of HIPEC remains investigational. Most analyses suggest a potential benefit in patients with platinum-sensitive recurrence, but the evidence is limited and highly heterogeneous. Tumor biology in recurrent cases is often characterized by increased chemoresistance and prior surgical interventions, which may reduce HIPEC efficacy [13]. HIPEC itself has not been used in Indonesia and several countries, especially in ovarian cancer, due to considerations regarding potential, safety, cost, and equipment maintenance. Therefore, this literature review aims to determine the potential of HIPEC as a modality of chemotherapy therapy in EOC patients compared with conventional chemotherapy therapy.

## Methods

### Study design

This meta-analysis and systematic review design was prepared based on the PRISMA framework and Cochrane Handbook for Systematic Reviews of Interventions version 6.3, 2022 [14, 15]. This study was registered in PROSPERO with registration number CRD42024579266.

### Search strategy

The data used in this review came from the Scopus, ProQuest, Taylor & Francis, Cochrane, EBSCO, and PubMed databases. This literature review uses data obtained on May 3, 2023, using Boolean operators to obtain a total of 15 valid and reliable journals based on predetermined criteria (Table 1).

**Table 1:** Literature search keywords

Database	Keywords
Pubmed, Taylor and Francis, Scopus, EBSCO, Cochrane, and Proquest	(“Cytoreductive surgery” OR “CRS”) AND (“HIPEC” OR “Hyperthermic Intraperitoneal Chemotherapy”) AND (“ovarian cancer” OR “epithelial ovarian cancer” OR “EOC”) AND (“primary” OR “recurrent”)

### Study eligibility criteria

Inclusion and exclusion criteria were determined to ensure the data were specific and relevant before the literature search. Inclusion criteria included (1) clinical trial studies using RCTs, (2) studies published in the last ten years, (3) AEs patients, (4) peer-reviewed journals, (5) studies with HIPEC intervention, and (6) outcome studies measured by Progression-free survival (PFS), overall survival (OS), AEs, and cost-effectiveness. Meanwhile, the exclusion criteria consisted of (1) incomplete text articles, (2) language incompatibility, and (3) incomplete reporting of results. Furthermore, articles that were not available online, as well as those focusing on preclinical or in vivo research, were excluded. The authors independently assessed each study for eligibility, resolving any disagreements through discussion and mutual agreement. Inclusion criteria were based on the PICOS framework, as listed in Table 2.

**Table 2:** Inclusion criteria based on PICOS

Population	Intervention	Comparison	Outcome	Study design
Epithelial ovarian cancer patients	*Cytoreductive Surgery* + *Hyperthermic Intraperitoneal Surgery*	*Cytoreductive surgery, chemotherapy, surgery, debulking surgery,* standard therapy	*Progression-free survival* (PFS)*, overall survival* (OS), adverse event, and *cost effectiveness*	Cross-sectional, case control, cohort, randomized controlled trials

### Data extraction

The data extracted from the studies included in this literature review consisted of (1) author and year of publication, (2) study characteristics and setting, (3) study population including sample size and subject type, (4) intervention and control including duration of administration and *follow-up*, and (5) study outcomes. Study characteristics were conducted by four reviewers (VEA, LGL, IMRDP, and NDPG) evaluating the study qualitatively, while another authors (AJP and AAM) double-checked the retrieved data for use in the literature.

### Quality assessment and publication bias

The risk of bias (RoB) was analyzed using the Revised Tool RoB 2.0, which consists of five domains for studies that are *randomized controlled trials* (RCTs) [16]. The results of the analysis will then be recorded in the bias domain file (.xlsx) and uploaded on the ROBVIS website to get graphic results. For cohort and *case–control* studies, study quality assessment was carried out using the *Newcastle–Ottawa Scale* (NOS) which consists of three domains and was recorded in a domain bias file (.xlsx) before being uploaded in the article file (.docs) [17]. The overall potential risk of NOS is based on the results of stars each domain with the following details: 0–3 stars is high risk, 4–6 is moderate risk, and 7–9 is low risk of bias. Journal quality analysis was conducted by the four reviewers (VEA, LGL, IMRDP, and NDPG) separately, and disagreements were resolved between reviewers and consult to two reviewers (AAM and AJP).

### Quantitative data analysis

Statistical analysis was performed using Review Manager 5.4.1 (The Nordic Cochrane Center, The Cochrane Collaboration, Copenhagen). The elements extracted from the studies were median months of follow-up, hazard ratio, confidence interval, and *p*-values of overall survival and progression-free survival of both intervention and control groups after treatment. In addition, some adverse events were extracted for both groups. After that, generic inverse variance (GIV) type analysis and pooled randomized effect model were used to interpret and calculate the impact through log [hazard ratio]. Results are presented in forest plots to assess heterogeneity, accumulated outcomes, and publication bias and funnel plots to observe outliers with 95% confidence intervals (CIs). The main findings that guided the reviewers in the statistical analysis were OS and PFS by comparing HR and median months of follow-up in both groups and adverse events by analyzing OR in both groups. Subgroup analysis for OS and PFS outcomes was performed based on the type of EOC, distinguishing between primary and recurrent cases. Meanwhile, in the analysis of AEs between the CRS + HIPEC and the control group, subgroup analysis was conducted based on the patients’ signs and symptoms. The *I*^2^ statistic was used to analyze heterogeneity, with cut-off criteria of 0–25% (not significant), 26–50% (low), 51–75% (medium), and 76–100% (high) [14]. The Duval and Tweedie trim-and-fill method was used to analyze outlier data in the study when heterogeneity was found to be high.

## Results

### Search results and study characteristics

**Fig. 1:**
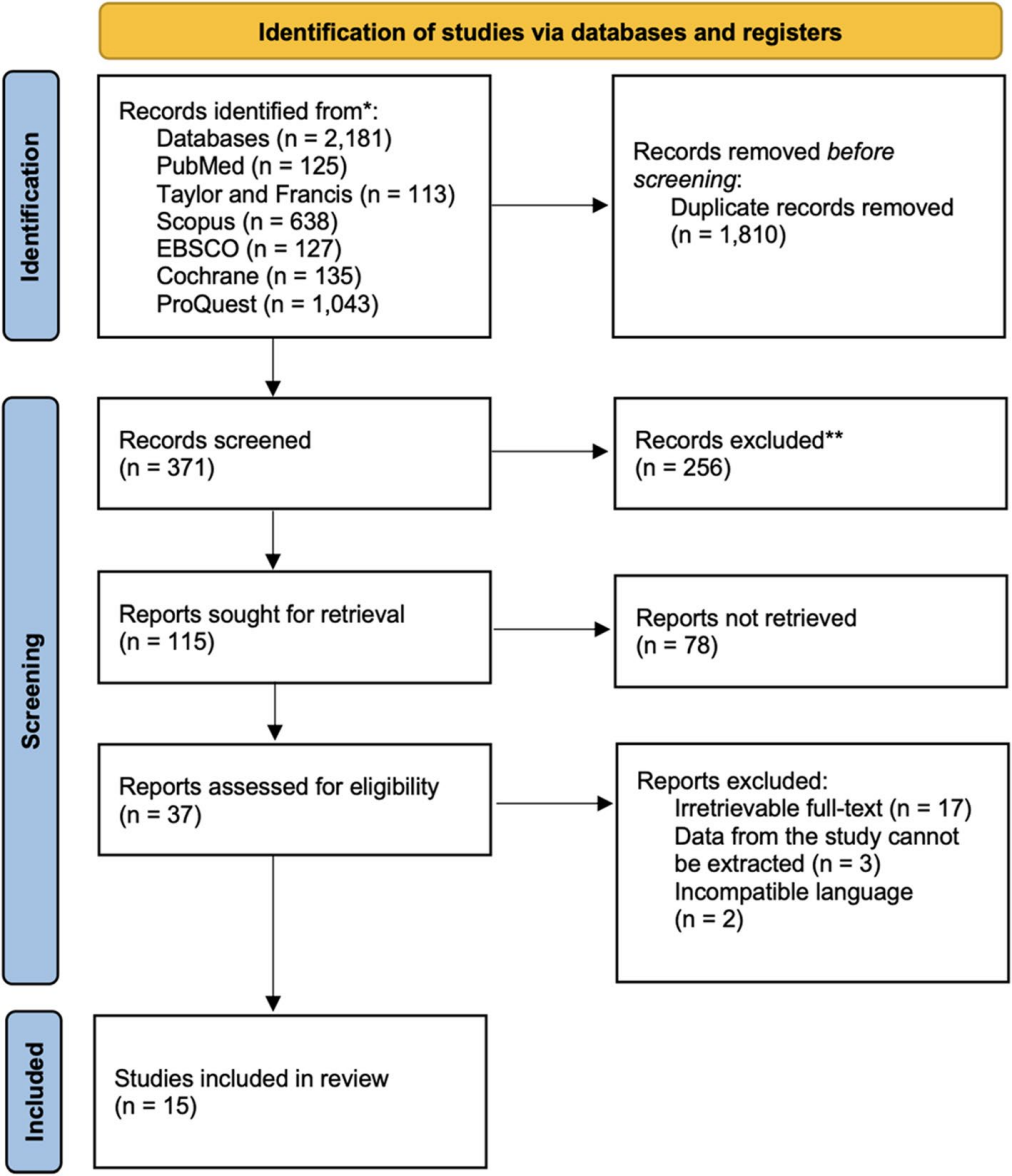
PRISMA schematic diagram for the screening and selection of eligible studies

The database serach, consisiting of Scopus, ProQuest, Taylor & Francis, Cochrane, EBSCO, and PubMed, yielded a total 2181 articles. The articles were exported and followed by duplication removal. Four independent authors (VEA, LGL, IMRDP, and NDPG) screened articles by going through the title and abstracts. Following a screening of titles and abstracts, 2166 studies were excluded. Finally, 15 studies were included for qualitative analysis consisting three observational studies and three randomized controlled trials. Out of six studies, four studies were included for quantitative synthesis consisting 5 RCT studies, 6 cohort studies, and 4 case–control studies with a total of 1982 participants consisting of an intervention group and a control group. The screening and selection process is summarized in the PRISMA schematic diagram in Fig. 1. The studies were conducted in multiple countries (Greece, France, Brazil, China, South Korea, Netherlands, Spain, Italy, and USA) with the intervention in the use of CRS + HIPEC while the control used CRS alone. They were further randomized into intervention groups, control groups, or multiple intervention groups for follow-up. Outcomes were measured using OS, PFS, and AEs including leukopenia, neutropenia, nausea, electrolyte, and disorders. Detailed characteristics of the included studies and study output are listed in Tables 3 and 4. A cost-utility analysis was performed by narrative review of 4 studies using Incremental Cost-Effectiveness Ratio (ICER), Quality-Adjusted Life Years (QALY), and Willingness to Pay (WTP) parameters as listed in Table 5.

**Table 3:** Study characteristics

Author	Research design	Year	Country	Number of patients (HIPEC/ control)	Age	Primary recurrent	Stadium	Results	NACT	Surgery	Postoperative chemotherapy	PCI score	Cytoreductive surgery	HIPEC regimen	HIPEC temperature	HIPEC duration	HIPEC frequency
Cascales-Campos et al. [18]	Retrospective cohort	2014	Spain	52/35	57 (29–73)	Primary	IIIc–IV	PFS/AE	Platinum and taxanes, 3–4 cycles	PDS/IDS	Platinum and taxanes, 6–8 cycles	9 (3–26)/6 (3–16), *p* < 0.01	NR	Paklitaxel (60 mg/m^2^)	42 °C	90 min	1
Antonio et al. [19]	RCT	2022	Spain	35/36	56/65.5	Primary	IIIb–IIIc	DFS/OS/QoL/Morbidity	Carboplatin (AUC 5) and paclitaxel (175 mg/m^2^), at least 3 cycles	PDS/IDS	Carboplatin (AUC 5) and paclitaxel (175 mg/m2), maximum 6 cycles	7 (2–29)/10 (2–22), *p* > 0.005	CC0: 32 (88.9)/33 (94.3); CC1: 4 (11.1)/2 (5.7)	Sisplatin 75 mg/m^2^	42 °C	60 min	1
Ceresoli et al. [20]	Retrospective case control	2018	Italy	28/28	58.99/61.55	Primary	IIIc–IV	OS/PFS/AE	Carboplatin and paklitaxel	IDS	NR	8.25 (6.79)/6.36 (6.19)	CC0: 23 (92.9)/23 (92.9); CC1: 1 (3.6)/1 (3.6)	Sisplatin (100 mg/m^2^) and paklitaxel (175 mg/m^2^)	41.5 °C	90 min	1
Charo et al. [21]	Retrospective cohort	2020	USA	20/48	21–83	Primary	IIIc–IVb	OS/PFS/AE	Carboplatin and paklitaxel; carboplatin and taxotere; carboplatin, paklitaxel and bevacizumab; other, 3–6 cycles	IDS	Carboplatin and paclitaxel; carboplatin and taxotre; carboplatin, paklitaxel, and bevacizumab; others, 3–6 cycles	17.5 (17.9), 0–34/N/A	CC0 - 1: 20/36 (75.0%); CC2: 0/12 (25.0%)	NR	NR	NR	NR
Lei et al. [22]	Retrospective cohort	2020	China	425/159	55.0 (10.5)	Primary	III	OS/AE	NR	PDS	NR	NR	Residual mass 1 cm 312 (73.4)/109 (68.6); > 1 cm 113 (26.6)/50 (31.4)	Cisplatin (50 mg/m^2^)	43 °C	60 min	SD = 2.8 (0.8)
Mendivil et al. [23]	Retrospective case control	2017	USA	69/69	59.8 (11.3)/62.9 (10.5)	Primary	IIIa–IV	OS/PFS	11 (15.9)/17 (24.6)	PDS/IDS	Paclitaxel (80 mg/m^2^) and carboplatin (AUC 6), 6 cycles	NR	Optimal cytoreduction 69/64 (92.7); Suboptimal 0/5 (7.3)	Carboplatin (AUC 10)	41.5 °C	90 min	1
van Driel et al. [24]	RCT	2018	Netherlands	122/123	61 (55–66)/63 (56–66)	Primary	III	OS/PFS/AE	Carboplatin (AUC 5–6) and paclitaxel (175 mg/m^2^), 3 cycles	IDS	Carboplatin and paclitaxel, 3 cycles	NR	R1-R1: 119 (98%)/120 (98%)	Sisplatin (100 mg/m^2^)	40 °C	90 min	1
Lim et al. [25]	RCT	2022	South Korea	92/92	52.0 (46–59.5)/53.5	Primary	III–IV	OS/PFS/AE	Carboplatin (AUC 5) and paclitaxel (175 mg/m^2^), 3 cycles	PDS	Paclitaxel and carboplatin, 6 cycles	0–5, 22 (23.9)/29 (31.5); 6–10, 70 (76.1)/63 (68.5)	Residual tumor < 1 cm	Sisplatin (75 mg/m^2^)	41.5 °C	90 min	1
										IDS							
Gruner et al. [26]	Retrospective cohort	2021	America	21/40	63.1 ± 9.2/66.3 ± 9.5	Primary	III–IV	PFS/EA	NR	IDS	NR	NR	Residual tumor < 1 cm	NR	NR	NR	NR
He et al. [27]	Retrospective case control	2021	China	121/76	58.38 (10.89)/55.20 (10.87	Primary	III	OS/PFS/AE	Carboplatin (AUC 5–6) and paclitaxel (175 mg/m^2^), 2–4 cycles	IDS	Paclitaxel (175 mg/m^2^) and carboplatin (AUC 5–6), 3–4 cycles	NR	Optimal (R0 + R1) 113 (93.4)/70 (92.1); sub-optimal (residual disease > 1 cm) 8 (6.6)/6 (7.9)	Sisplatin (60 mg/m^2^)	42–43 °C	60 min	3 (1/3/5 day)
Baiocchi et al. [28]	Retrospective cohort	2016	Brazil	29/50	30–80	Recurrent	III–IV	OS/PFS	NR	SCR	NR	< = 6, 15 (51.7)/24 (53.3); > 6, 14 (48.3)/21 (46.7); missing 0/5	CC0 - 1 27: (93.1)/42 (87.5); CC2 - 3: 2 (6.8)/6 (12.5); Lost 0/2	Mithomycin C (10 mg/m^2^) and cisplatin (50 mg/m^2^)/cisplatin (50 mg/m^2^) and doxorubicin/cisplatin (50 mg/m^2^)/oxaliplatin	41–42 °C	More than 90 min	1
Cascales-Campos et al. [29]	Retrospective cohort	2015	Spain	32/22	56.4 ± 10.8/57.4 ± 9.7	Recurrent	I–II 7/9; III–IV 25/13	PFS/AE	NR	SCR	Paklitaxel (175 mg/m^2^) and carboplatin (AUC 6), 6 cycles	8 (2–23)/4 (2–16), *p* = 0.001	NR	Paklitaxel (60 mg/m^2^)	42 °C	90 min	1
Le Brun et al. [30]	Retrospective case control	2014	France	23/19	> 55 years 16/13	Recurrent	I–II 2/1; III–IV 21/18	OS	Platinum-based second-line chemotherapy, 6 cycles	SCR	NR	NR	Complete secondary surgery, CC0	Sisplatin, eloxatin, or mitomycin	42 °C	Sisplatin 60 min, Eloxatin and mitomycin 30 min	1
Spiliotis et al. [31]	RCT	2015	Greece	60/60	58.3/58.1	Recurrent	IIIc 41/35; IV 19/25	OS	NR	SCR	NR	< 5, 7 (11.7%)/8 (13.3%); 5, < 10, 24 (40%)/22(36.7%); 10, 29 (48.3%)/30 (50%)	CC0 39 (65%)/33 (55%); CC1 12 (20%)/20 (33.3%); CC2 9 (15%)/7 (11.7%)	PS sisplatin (100 mg/m2) and paklitaxel (175 mg/m2); PR doxorubicin (35 mg/m2) and paklitaxel (175 mg/m^2^)/mitomycin (15 mg/m^2^)	42.5 °C	60 min	1
Zivanovic et al. [32]	RCT	2021	America	49/49	NR	Recurrent	NR	PFS/OS	NR	SCR	Carboplatin-based chemotherapy	NR	NR	Carboplatin (800 mg/m^2^)	NR	90 min	1

*Abbreviation* *RCT* randomized controlled trials, *HIPEC* hyperthermic intraperitoneal chemotherapy, *PFS* progression-free survival, *AE* adverse events, *DFS* disease-free survival, *OS* overall survival, *QoL* quality of life, *NACT* neoadjuvant chemotherapy, *AUC* area under the curve, *PDS* primary debulking surgery, *IDS* interval debulking surgery, *SCR* secondary cytoreductive surgery, *PCI* peritoneal cancer index, *CC* complete cytoreduction, *SD* standard deviation, *NR* not reported

**Table 4:** Study outputs

Author	**OS**				**PFS**				**Leukopenia**		**Neutropenia**		**Nausea**		**Electrolyte disorders**	
	CRS (months)	HIPEC (months)	HR	CI	CRS (months)	HIPEC (months)	HR	CI	CRS	HIPEC	CRS	HIPEC	CRS	HIPEC	CRS	HIPEC
Cascales-Campos et al. [18]	NR	NR	NR	NR	26.7 ± 3.2 (mean DFS, not median)	39.1 ± 2.9 (mean DFS, not median)	8.77 on non-HIPEC	2.76–14.42 on non-HIPEC	NR	NR	NR	NR	NR	NR	NR	NR
Antonio et al. [19]	45	52	0.12	0.02–0.89	12 (DFS, not PFS)	18 (DFS, not PFS)	0.12	0.02–0.89	NR, the most common aAEt in the CRS group was ileus (9/36), and in the HIPEC group was anemia (5/35)							
Ceresoli et al. [20]	32.53	No median reached	NR	NR	13.23 (DFS, not PFS)	13.96 (DFS, not PFS)	1.39	0.70–2.75	- Major complications (no examples mentioned) occurred in CRS (5/28) and HIPEC (7/28). 1 patient in the HIPEC group developed acute renal failure. Recurrence in CRS was most common at the peritoneal site (10/28), while HIPEC was systemic (13/28)							
Charo et al. [21]	73–105	73–105	0.67	0.48–0.94	NR	NR	NR	NR	NR	NR	NR	NR	NR	NR	NR	NR
Lei et al. [22]	34	49.8	0.63	0.49–0.82	NR	NR	NR	NR	72/159	252/425	74/159	249/425	81/159	299/425	91/159	373/425
Mendivil et al. [23]	33.6 (mean)	33.8 (mean)	NR	NR	20 (mean)	25.1 (mean)	2.1028	1.2941–3.4167	NR	NR	NR	NR	20/138		NR	NR
van Driel et al. [24]	33.9	45.7	0.67	0.48–0.94	10.7	14.2	0.66	0.50–0.87	NR	NR	NR	NR	73/122	76/118	7/122	10/118
Lim et al. [25]	NR	71.3	0.53	0.29–0.96	23.9	29.7	0.6	0.37–0.99	62/92	67/92	53/92	63/92	63/92	68/92	92/92	92/92
	48.2	61.8			15.4	17.4										
Gruner et al. [26]	11.4	13	0.69	0.36–1.38	Not estimable	32.1	0.69	0.35–1.35	NR, explained that HIPEC had no impact on increasing the risk of anastomotic leakage during IDS with colorectal resection and reanastomosis, the most common adverse events were surgical site infection (SSI) in HIPEC (3/21), without HIPEC (4/40)							
He et al. [27]	40	51	0.52	0.35–0.78	19	24	0.46	0.33–0.65	2/76	21/121	NI	NI	15/76	27/121	20/76	38/121
Baiocchi et al. [28]	NR	NR	1.35	0.59–3.08	NR	NR	1.08	0.60–1.93	NR	NR	NR	NR	NR	NR	NR	NR
Cascales-Campos et al. [29]	NR	NR	NR	NR	77 (DFS, not PFS)	77 (DFS, not PFS)	2.14 on non-HIPEC	0.86–4.21 on non-HIPEC	NR	NR	NR	NR	NR	NR	NR	NR
Le Brun et al. [30]	NR	NR	0.15	0.03–0.66	NR	23.9 (DFS, not PFS)	NR	NR	NR	NR	NR	NR	NR	NR	NR	NR
Spiliotis et al. [31]	13.4	26.7 (sensitive 26.8, resistant 26.6)	1.41	0.36–5.51	NR	NR	NR	NR	NR	NR	NR	NR	NR	NR	NR	NR
Zivanovic et al. [32]	59.7	52.5	1.39	0.73–2.67	15.7	12.3	1.54	1–2.37	NR	NR	NR	NR	0/49	1/49	NR	NR

*Abbreviation* *HIPEC* hyperthermic intraperitoneal chemotherapy, *PFS* progression-free survival, *AE* adverse events, *DFS* disease-free survival, *OS* overall survival, *NR* not reported

**Table 5:** Cost feasibility analysis

Study	Country	Grade	Cost		Incremental Cost	QALY		Incremental QALY	ICER/ QALY	WTP/ QALY
		Stage	ICRS	ICRS + HIPEC		ICRS	ICRS + HIPEC			
Koole et al., 2019 [8]	Netherlands	III	€70,046	€85,791	€15,000	2.12	2.68	0.56	€28,299	€80,000
Lim et al., 2019 [1]	USA	III	$78,849	$79,954	$1105	2.9	2.45	0.45	$2436	$50,000
Kim JH et al., 2023 [9]	Korea	III-IV	$16,460	$19,060	$2600	7.16	10.8	3.64	$708.3	$30,496
Penn et al., 2022	USA	III	$32,169	$38,405	$6236	NR	NR	1.3	$7854	NR

*Abbreviation* *ICRS* iterative cytoreductive surgery, *QALY* quality-adjusted life year, *HIPEC* hyperthermic intraperitoneal chemotherapy, *ICER* incremental cost- effectiveness ratio, *WTP* willingness to pay, *NR* not reported

### Qualitative assessment

**Fig. 2:**
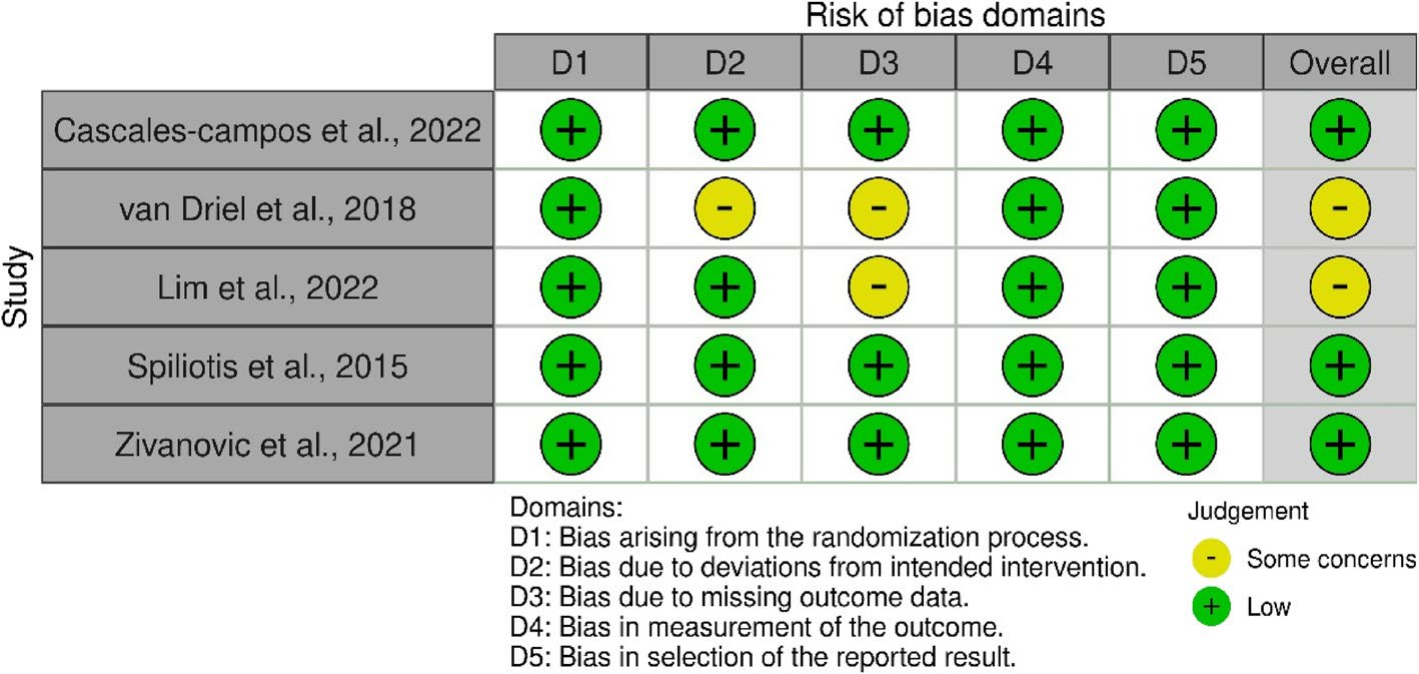
Results of risk of bias quality assessment using the risk of bias (RoB) 2.0 for randomized controlled trials included in this systematic review and meta-analysis

**Fig. 3:**
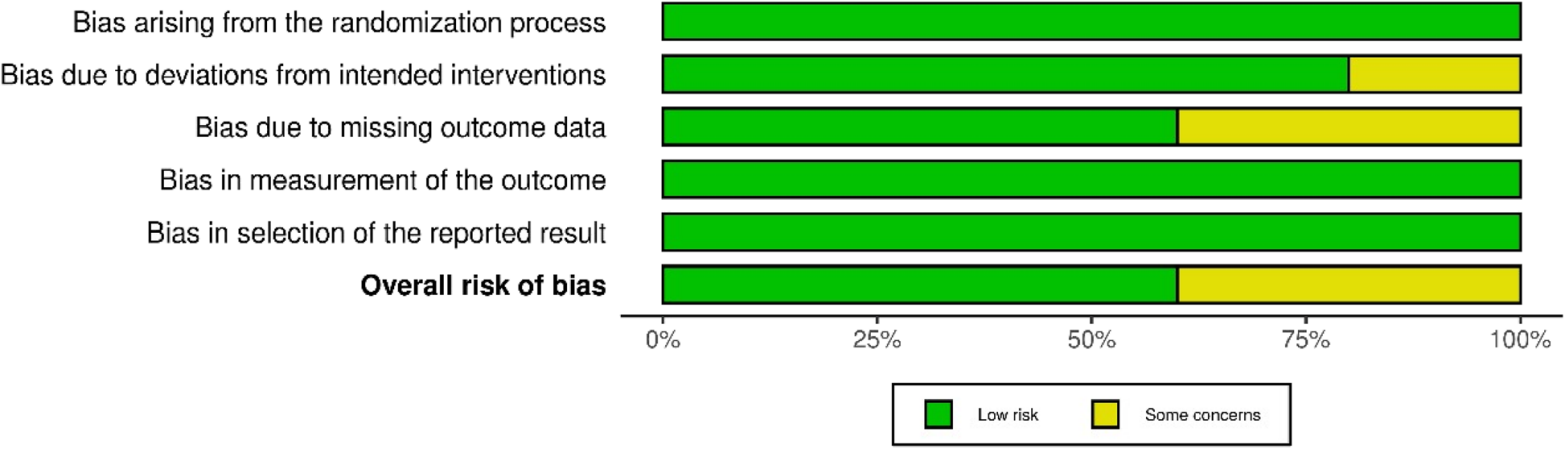
Percentage of the quality assessment according to the five domains using RoB- 2 tools from cochrane

Qualitative analysis was performed on all studies with 9 observational analytic studies assessed for quality using NOS, while five RCT studies were analyzed using RoB 2.0. The risk of bias (RoB) 2.0 quality assessment was employed to assess the quality of the included randomized controlled trials, where the results are presented in (Figs. 2 and 3). Three studies are of low risk of bias, while two studies considered a moderate risk of bias under all domains except the bias due to missing outcome data with only 60% being low risk [19, 24, 25, 31, 32]. The risk of bias itself assessed five different domains, including the bias arising from the randomization process, bias due to deviations from intended interventions, bias due to missing outcome data, bias in measurements outcome, and bias in the selection of the reported result.

The risk of bias quality assessment was employed to assess the quality of the included observational study trials, where the results are presented in Tables 6 and 7. Two studies are of low risk of bias, while the other one was moderate risk of bias under all domains with only 66% being low risk. The risk of bias itself assessed three different domains, including the selection domain, comparison domain, and outcome domain.

**Table 6:** Newcastle–Ottawa Scale (NOS) analysis results from cohort studies

No	Study name	Selection domain^a^	Comparison domain^b^	Outcome domain^c^	Potential risk habits
1	Cascales-Campos et al., 2014 [18]	***	**	***	Low
2	Charo et al., 2020 [21]	****	**	***	Low
3	Lei et al., 2020 [22]	****	*	***	Low
4	Gruner et al., 2021 [26]	****	**	***	Low
5	Baiocchi et al., 2016 [28]	****	**	***	Low
6	Cascales-Campos et al., 2015 [29]	****	**	***	Low
7	Le Brun et al., 2014 [30]	****	**	***	Low

^a,b,c^An increase in asterisks (*) indicates better quality in the domain criteria being assessed

The maximum number of asterisks that can be assigned to each domain criterion is selection = 4, comparison = 2, and result = 3

**Table 7:** Newcastle–Ottawa Scale (NOS) analysis results from the case control study

No	Study name	Selection domain^a^	Comparison domain^b^	Outcome domain^c^	Potential risk habits
1	Ceresoli et al., 2018 [20]	****	*	**	Low
2	Mendivil et al., 2017 [23]	****	*	**	Low
3	He et al., 2021 [27]	****	*	***	Low

^a,b,c^An increase in asterisks (*) indicates better quality in the domain criteria being assessed

The maximum number of asterisks that can be assigned to each domain criterion is selection = 4, comparison = 2, and result = 3

### Efficacy of HIPEC on OS in patients with EOC

**Fig. 4:**
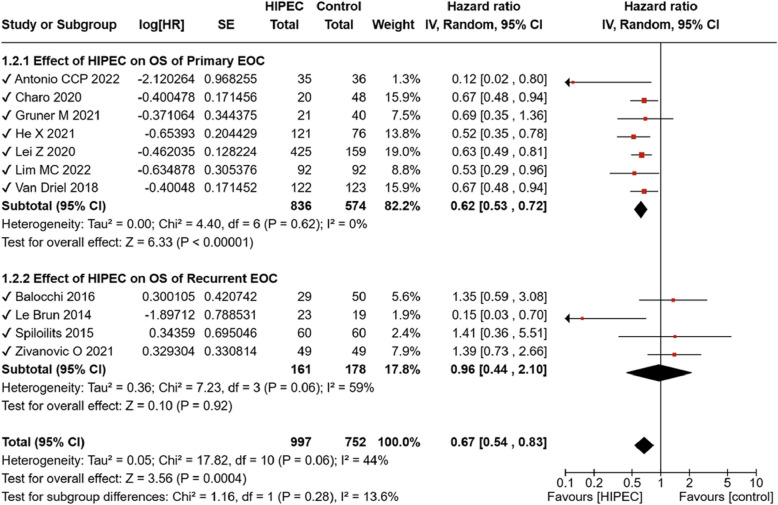
Forest plot of HIPEC vs. control based on OS

There were 11 studies that reported OS of intervention vs control with 1749 EOC patients (Fig. 4). The 11 studies were divided into two subgroups, namely between primary and recurrent EOC. Results were generally significant with *p* < 0.05 (*p* < 0.0004) and HR value 0.67 (95% CI, 0.54–0.83; *I*^2^ = 44%) with primary subgroup analysis *p* < 0.05 (*p* < 0.00001) and HR value 0.62 (95% CI, 0.53–0.72; *I*^2^ = 0%) and recurrent not significant *p* > 0.05 (*p* = 0.92) and HR value 0.96 (95% CI, 0.44–2.10; *I*^2^ = 59%). Thus, it can be concluded that HIPEC intervention can prolong OS duration in EOC patients with HR in each subgroup and overall being below*.*

**Fig. 5:**
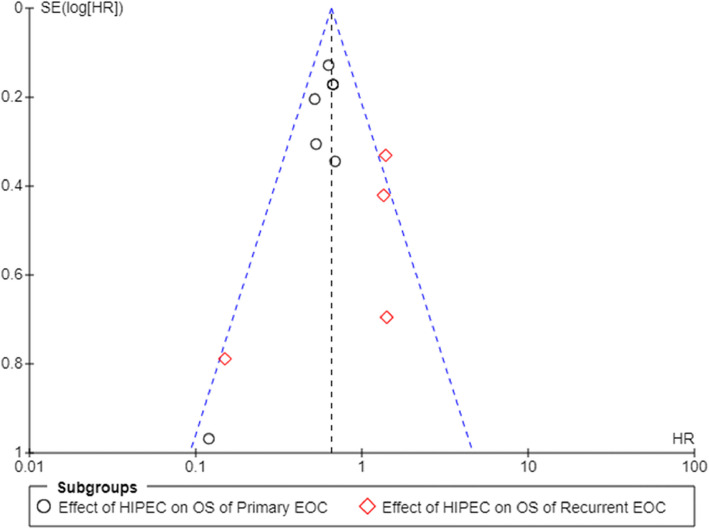
Funnel Plot of HIPEC vs. control based on OS

**Fig. 6:**
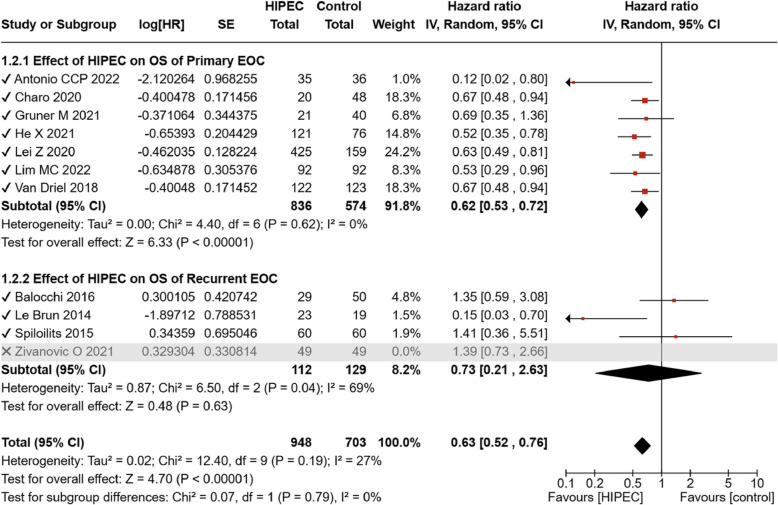
Forest plot with sensitivity analysis of HIPEC vs. control based on OS

Although heterogeneity is in the moderate category, which is *I*^2^ 44%, Duval and Tweedie’s trim-and-fill method for sensitivity analysis was used to identify outlier results in the study as shown in the funnel plot in Fig. 5. Outliers can be seen in Zivanovic’s (2021) study on recurrent EOC due to the possibility of post-operative chemotherapy using only carboplatin [32]. It was found that the total heterogeneity decreased to 27% with a significant improvement in the recurrent subgroup (*p* = 0.63) after its exclusion as listed in Fig. 6. The funnel plot shows asymmetry which could be an indication of publication bias in this analysis.

### Efficacy of HIPEC on PFS in patients with EOC

**Fig. 7:**
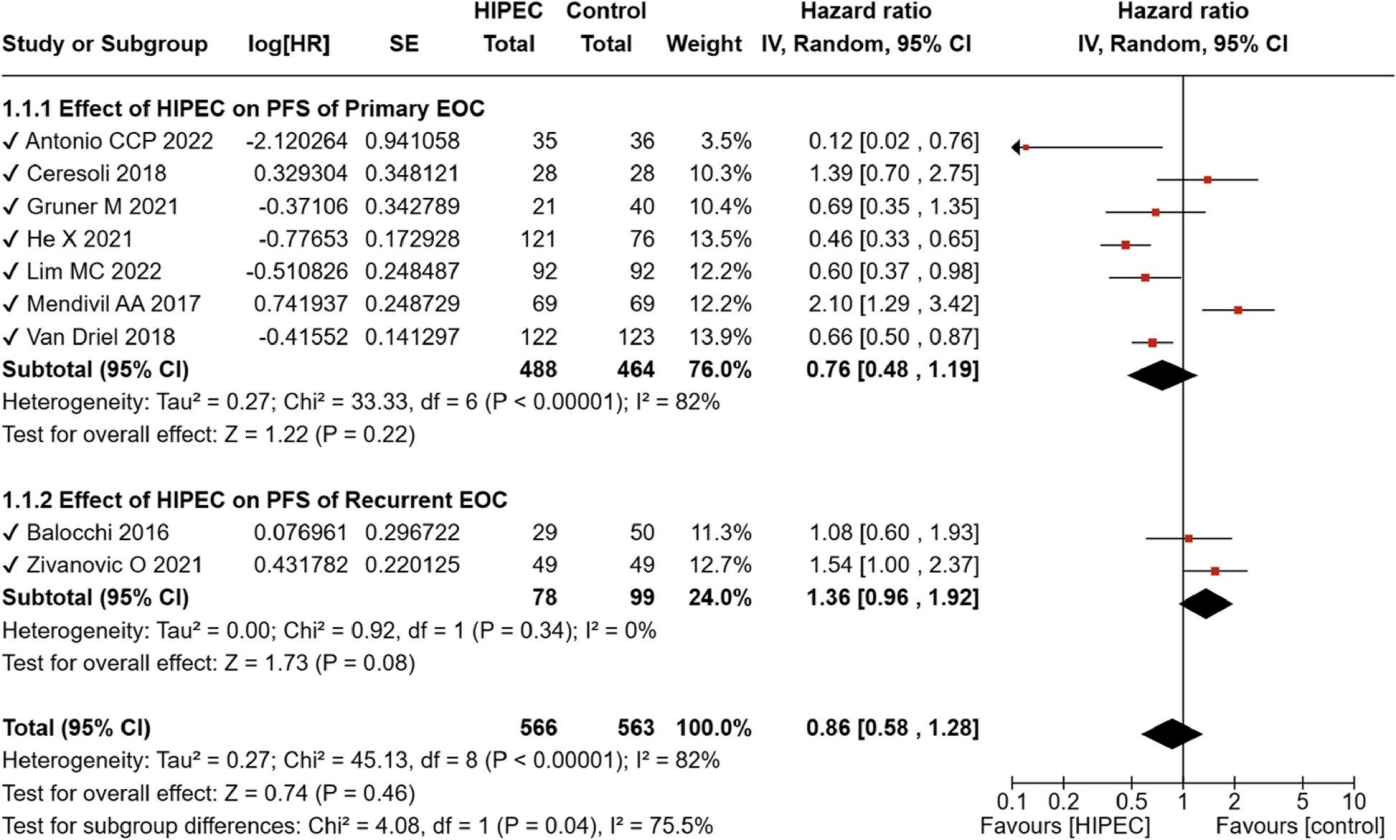
Forest Plot of HIPEC vs. control based on PFS

There were 9 studies that reported OS of intervention vs control with 1129 EOC patients (Fig. 7). The 11 studies were divided into two subgroups, namely between primary and recurrent EOC. Results were generally not significant with *p* > 0.05 (*p* = 0.46) and HR value 0.86 (95% CI, 0.58–1.23; *I*^2^ = 82%) with subgroup analysis of primary *p* > 0.05 (*p* = 0.22) and HR value 0.76 (95% CI, 0.48–1.19; *I*^2^ = 82%) and recurrent *p* > 0.05 (*p* = 0.08) and HR value 1.36 (95% CI, 0.96–1.92; *I*^2^ = 0%). Thus, it can be concluded that HIPEC can prolong the duration of PFS although with insignificant results.

**Fig. 8:**
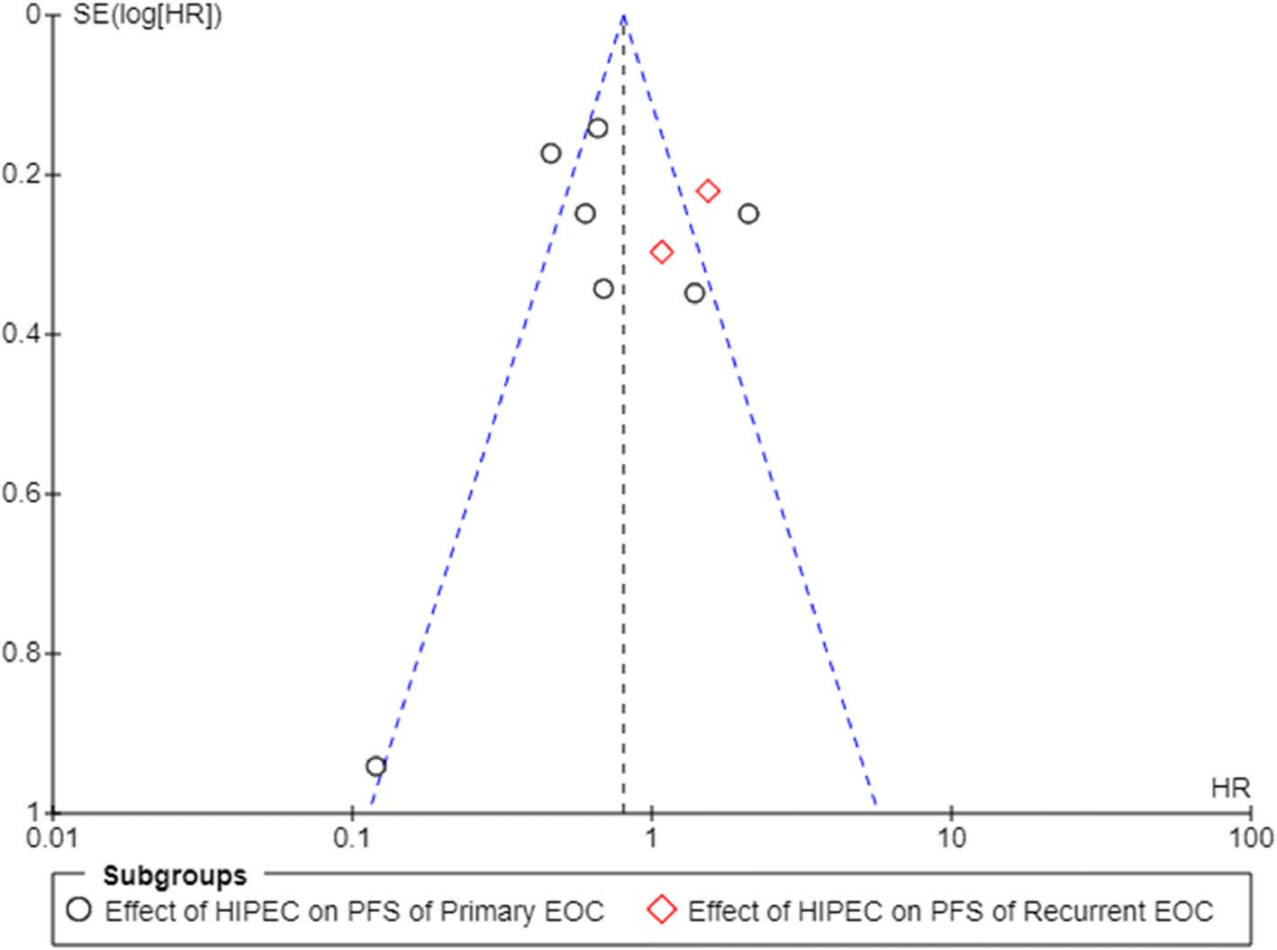
Funnel plot of HIPEC vs. control based on PFS

**Fig. 9:**
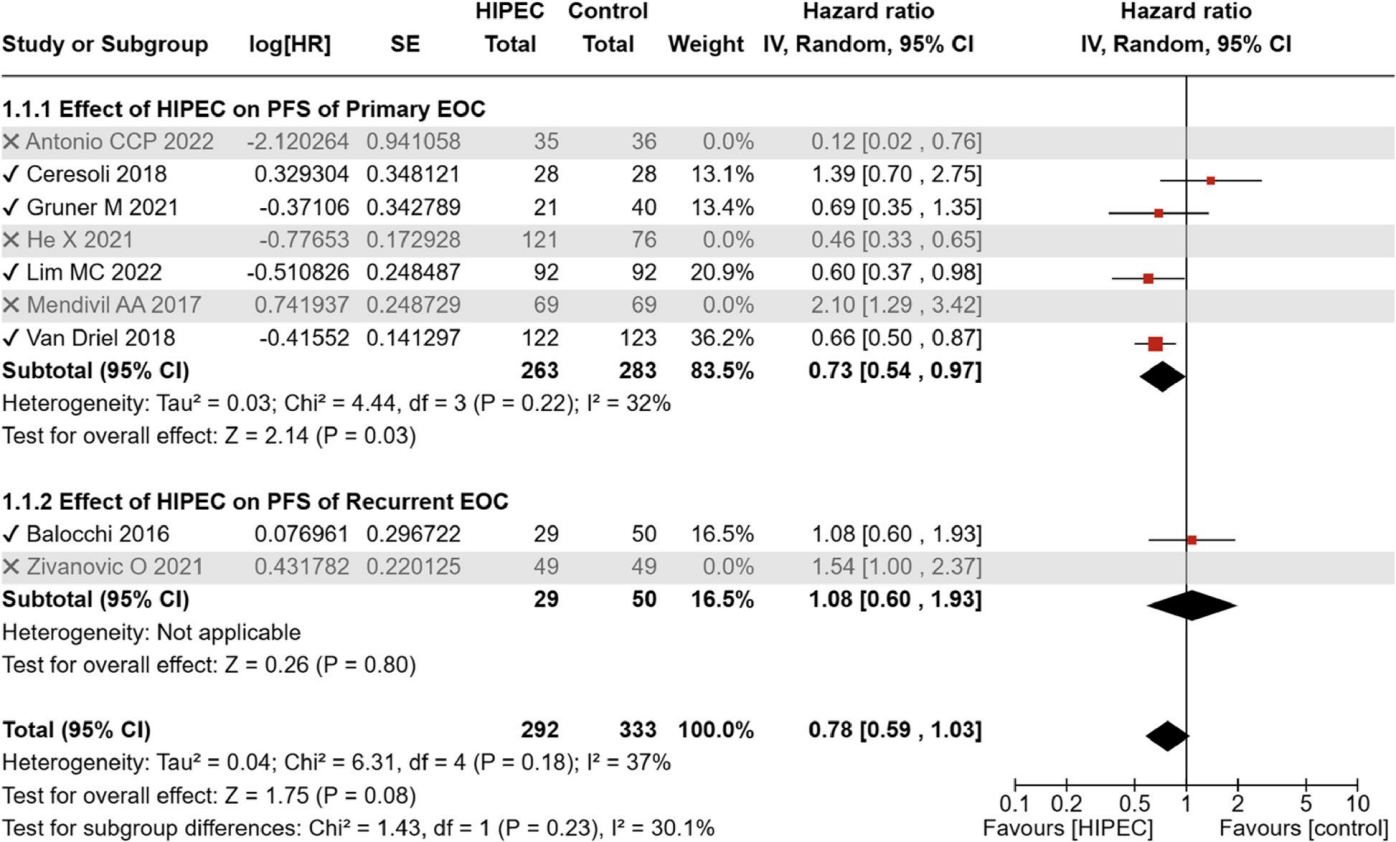
Forest plot with sensitivity analysis of HIPEC vs. control based on PFS

Despite the high heterogeneity of 82%, Duval and Tweedie’s trim-and-fill method for sensitivity analysis was used to identify outlier results in the study as shown in the funnel plot in Fig. 8. Outliers can be seen in the studies of Antonio (2022), He X (2021), and Mendivil (2017) in primary EOC and Zivanovic (2021) in recurrent EOC due to the difference in setting and the variety of surgical techniques, HIPEC regimens, and temperatures performed which contributed to the high level of heterogeneity observed in the analysis [19, 23, 27, 32]. After exclusion, there was an improvement in the overall results *p* > 0.05 (*p* = 0.08) and HR value 0.78 (95% CI, 0.59–1.03; *I*^2^ = 32%) with subgroup analysis of primary *p* < 0.05 (*p* = 0.03) and HR value 0.73 (95% CI, 0.54–0.97; *I*^2^ = 32%) and recurrent *p* > 0.05 (*p* = 0.80) and HR value 1.08 (95% CI, 0.60–1.93) as listed in Fig. 9. The funnel plot shows asymmetry which could be an indication of publication bias in this analysis.

**Fig. 10:**
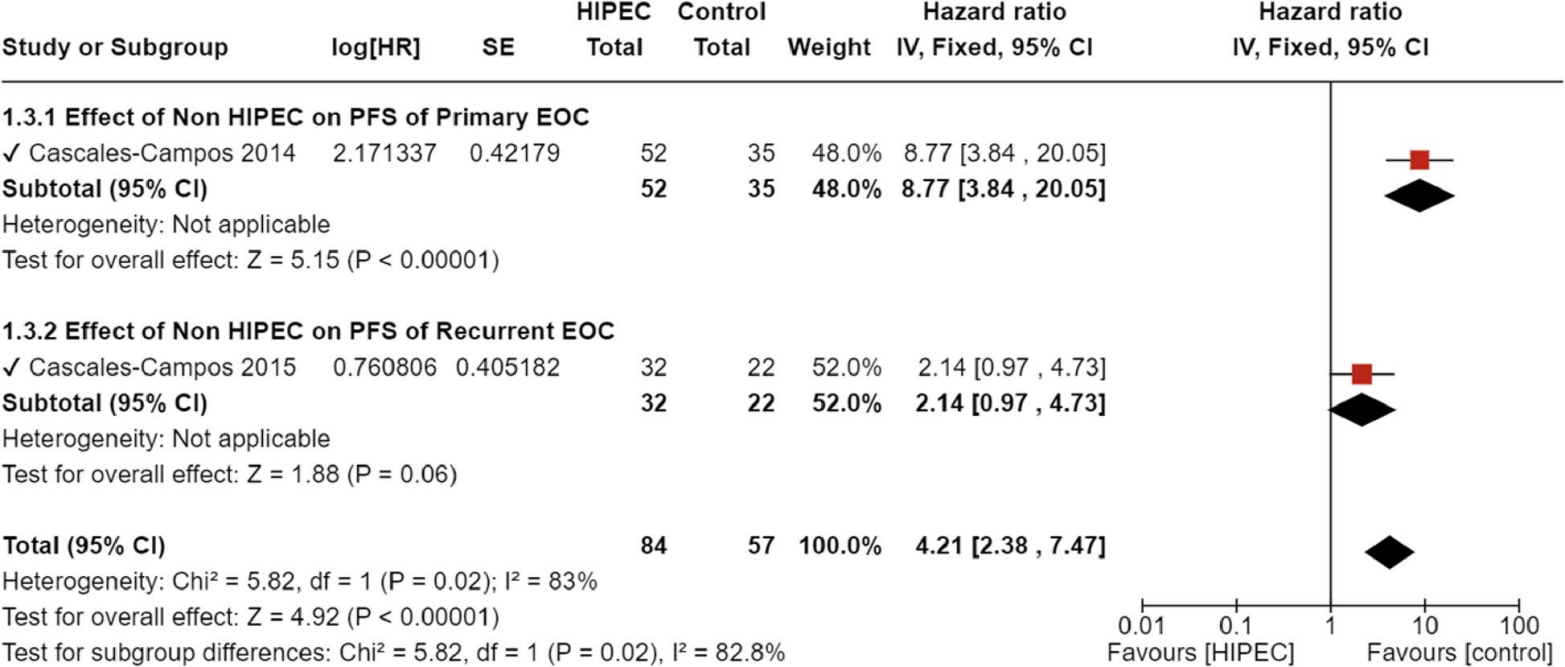
Forest plot of non-HIPEC vs. control based on PFS

There were also 2 studies that showed that in non-HIPEC as an intervention showed a significant decrease in overall PFS duration *p* < 0.05 (*p* < 0.00001) and HR value 4.21 (95% CI, 2.38–7.47) with primary subgroup analysis *p* < 0.05 (*p* < 0.00001) and HR value 8.77 (95% CI, 3.84–20.55) and recurrent *p* > 0.05 (*p* = 0.06) and HR value 2.14 (95% CI, 0.97–4.73; *I*^2^ = 0%) as listed in Fig. 10. Thus, it can be concluded that non-HIPEC can worsen PFS duration with HR value > 1 in each subgroup and overall value.

### Safety of HIPEC against AEs in patients with EOC

**Fig. 11:**
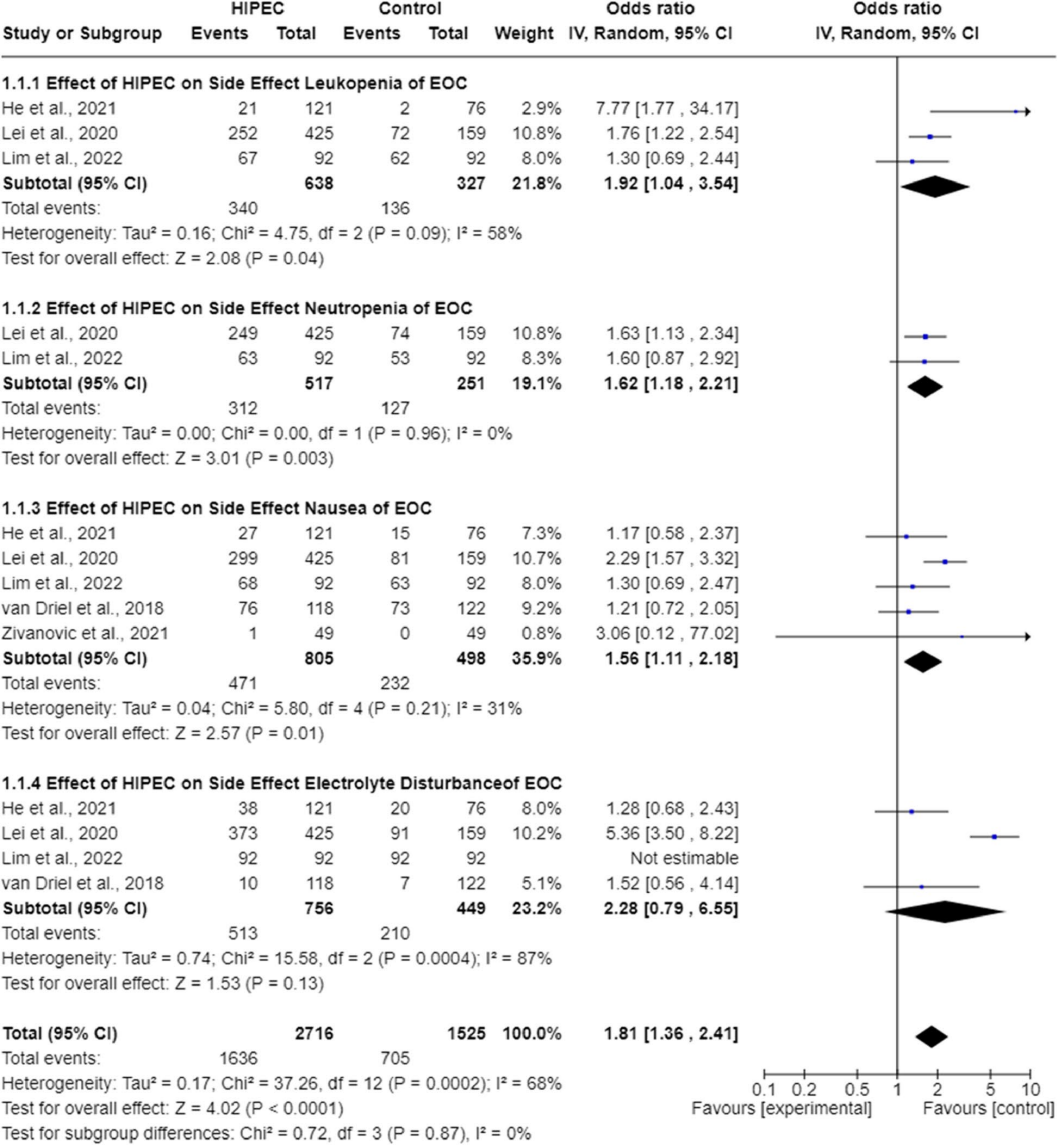
Forest plot of HIPEC vs. control by AEs

There were 5 studies that reported adverse events of intervention vs control. These 5 studies were divided into four subgroups, namely the incidence of leukopenia, neutropenia, nausea, and electrolyte imbalance (Fig. 11). The overall results suggest HIPEC were significantly increase an incidence of AEs compared to the control group with *p* < 0.05 (*p* < 0.0001) and OR 1.81 (95% CI, 1.36–2.41; *I*^2^ = 68%) with leukopenia subgroup analysis *p* < 0.05 (*p* = 0.04) and OR value 1.92 (95% CI, 1.04–3.54; *I*^2^ = 0%), neutropenia subgroup *p* < 0.05 (*p* = 0.003) and OR value 1.62 (95% CI, 1.18–2.21; *I* ^2^ = 0%), nausea subgroup *p* < 0.05 (*p* = 0.01) and OR value 1.56 (95% CI, 1.11–2.18; *I*^2^ = 31%), and electrolyte imbalance subgroup *p* > 0.05 (*p* = 0.13) and OR value 2.28 (95% CI, 0.79–6.55 *I*^2^ = 87%). Thus, it can be concluded that the control group did not cause AEs and it is a consideration for the use of HIPEC.

**Fig. 12:**
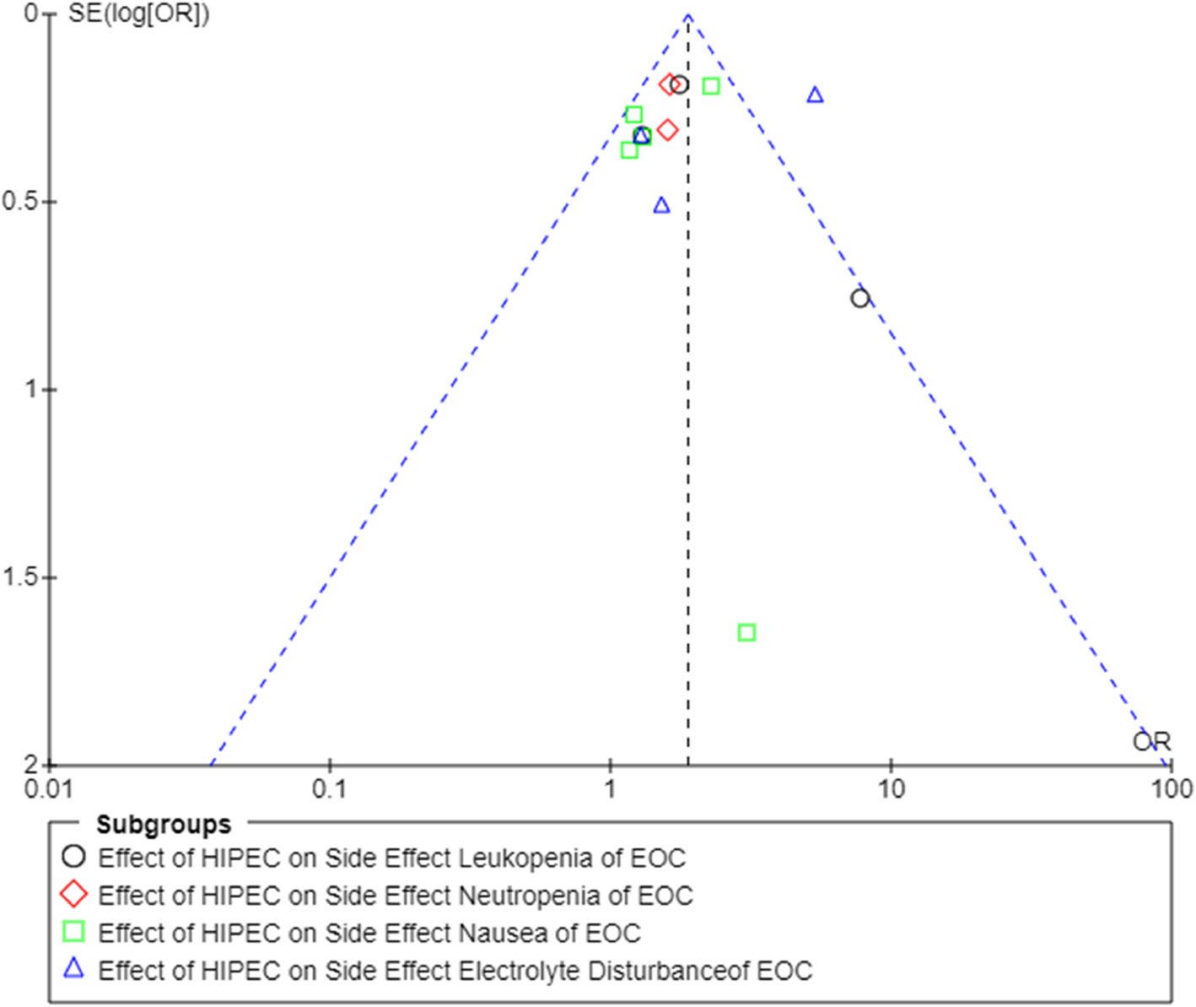
Funnel plot of HIPEC vs. control by AEs

**Fig. 13:**
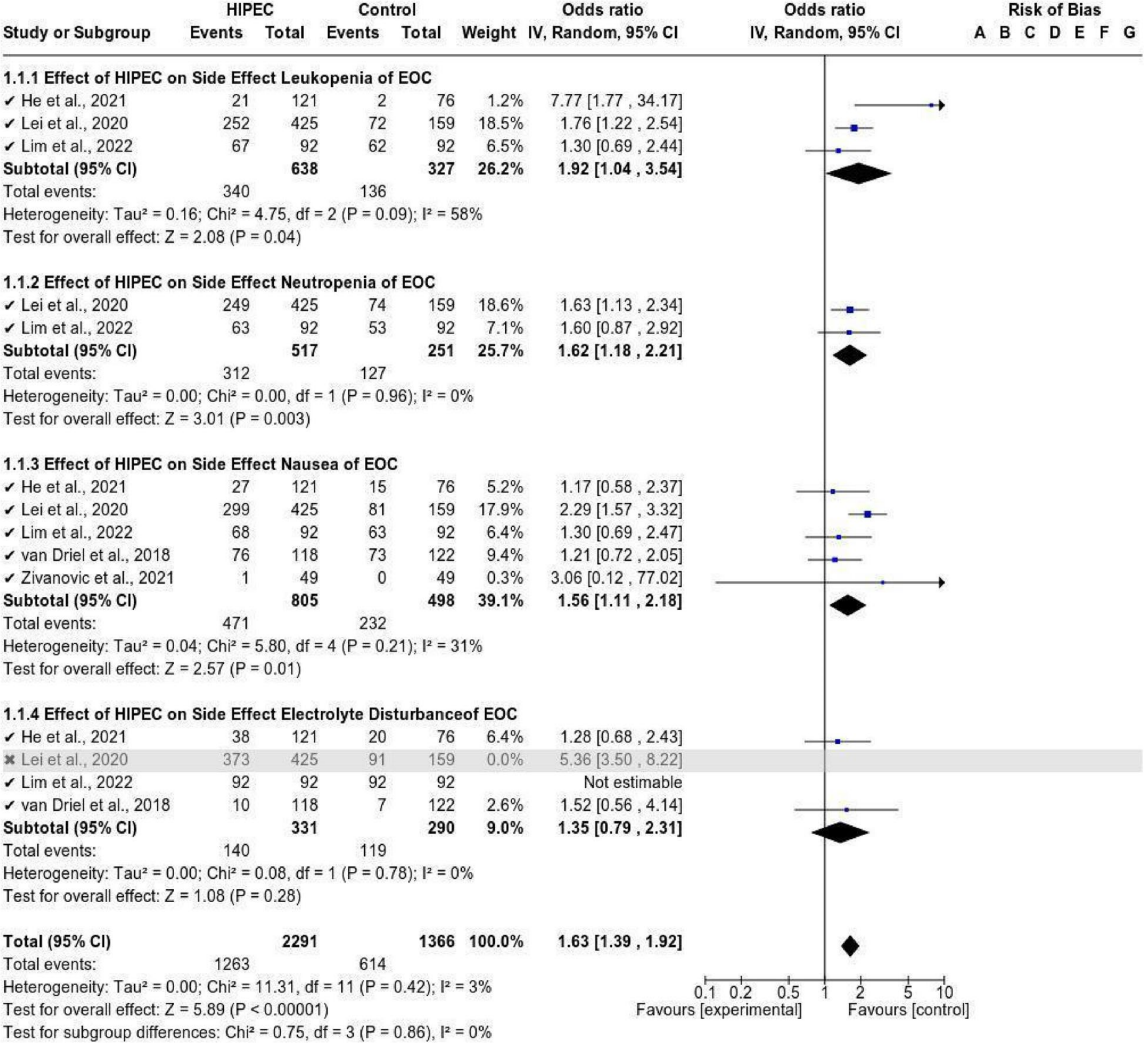
Forest plot with sensitivity analysis of HIPEC vs. control by AEs

Although heterogeneity is in the moderate category, namely I^2^ 68%, Duval and Tweedie’s trim-and-fill method for sensitivity analysis was used to identify outlier results in the study as shown in the funnel plot in Fig. 12. Outliers can be seen in Lei’s (2020) study on recurrent ovarian cancer which obtained a decrease in total heterogeneity to 3% and 0% in the electrolyte imbalance subgroup as listed in Fig. 13 [22]. The funnel plot shows asymmetry which can be an indication of publication bias in this analysis.

## Discussion

This systematic review and meta-analysis include 15 studies to evaluate the efficacy and safety of CRS + HIPEC in primary or recurrent EOC patient compared to control group. Involving 1982 participants, CRS + HIPEC showed the best value for extending OS and PFS, while for the AEs HIPEC no better than control group.

HIPEC is a post-cancer surgery procedure that involves administering chemotherapy intraperitoneally at a high temperature. It is also used to treat peritoneal metastases in various cancers, including ovarian cancer [33]. The method has attracted attention because high concentrations of drugs can be administered to the tumor without excessive systemic AEs [34]. HIPEC is performed after CRS to remove the probability that microscopic cancer cells may still remain. Beforehand, NACT is added to shrink the size of the tumor so that the CRS procedure can be easily performed. When the CRS procedure is complete, the abdominal cavity will be washed using sterile fluids to remove any remaining cancer cells [35]. In the HIPEC procedure, high doses of heated chemotherapy (saline solution 40–43 °C) are administered into the abdominal cavity to penetrate and destroy any remaining cancer cells. In this case, intraperitoneal chemotherapy allows the drug to reach high concentrations within the abdominal cavity, reducing the risk of systemic AEs and increasing its effectiveness through the mechanisms of convection, diffusion, and penetration of the drug into tissues [36]. During this procedure, probes are placed on both sides of the abdominal wall to accurately monitor the abdominal temperature. Furthermore, an inflow catheter is placed at the bottom of the abdomen, while an outflow catheter is placed at the top of the abdomen. Both catheters are connected to a perfusion machine that serves to warm, administer, and control the temperature and flow of the chemotherapy solution [36].

Analysis of the studies conducted showed that the provision of HIPEC intervention after CRS had a positive impact on EOC patients in the form of a significant increase in the length of time of patient survival or OS. The increase of HR (0.67) suggests a 33% lower risk of death with treatment. For example, if 30% of patients in the control group die within 2 years, an HR of 0.67 means about 20% would die with treatment. That is a 10% absolute risk reduction (ARR), meaning number needed of treat (NNT) is 10 patients to prevent one death over 2 years. This helps show the real-world value of the treatment, beyond just the statistical results. These results are in line with the results of a meta-analysis in a study conducted by Xia et al. [37] in which HIPEC is known to be able to improve the clinical prognosis of primary EOC in the form of increased OS values and decreased risk of intra-abdominal bleeding and constipation [37]. HIPEC can improve the outcome of CRS because the high temperature used in HIPEC can increase cell toxicity and fight resistance to platinum NACT [37]. The mechanism of intraperitoneal HIPEC intervention combined with hyperthermic conditions is also known to increase intracellular drug concentration with a more localized increase in concentration that can simultaneously minimize systemic toxicity compared to intravenous chemotherapy [28, 38]. The results of this study are also supported by a previous meta-analysis study by Cianci et al. [39] regarding the effect of HIPEC on patients with recurrent EOC which showed significant improvement in OS values in the first, second, third, and fifth years after intervention [39]. A case–control study conducted on 42 patients in France between 1997 and 2011 also resulted in a significant improvement in OS values through review in the fourth year after the intervention [30].

The observed funnel plot asymmetry suggests the presence of potential publication bias, which may have influenced the overall findings. Notably, the study by Zivanovic et al. appeared to contribute substantially to the heterogeneity and potential bias, likely due to its use of postoperative chemotherapy with carboplatin monotherapy [32]. In contrast, other included studies employed combination chemotherapy regimens, which may offer improved survival outcomes, thereby affecting the pooled estimates. However, the adjusted results have been obtained to show the pooled results with minimized bias. The success of HIPEC in improving OS outcomes also requires attention to the quality of CRS as a precursor to the intervention. Invasion rate, therapeutic success, and risk of complications are known to be influenced by the complexity and accuracy of previously performed CRS [38, 39].

The results of the meta-analysis through the forest plot data obtained in this study showed a positive correlation between the provision of HIPEC intervention and an increase in PFS value in EOC patients. The overall HR value was found to be 0.86 (HR < 1) which indicates an increase in the value of PFS in HIPEC intervention with a decrease in disease progressivity in the intervention group. The increase of HR (0.86) suggests a 14% lower risk of death with treatment. If 30% of patients in the control group die within 2 years, an HR of 0.67 means about 25.8% would die with treatment. This gives an ARR 4.2%, meaning number needed of treat (NTT) is 24 patients to prevent one death over 2 years. These results are supported by a randomized controlled trial (RCT) study conducted on 184 EOC patients with primary stages III and IV in South Korea which showed an increase in PFS duration in the HIPEC group for 1 month longer than the control group with a median follow-up of 69.4 months [25].

Although increasement was found, the results were insignificant with high heterogeneity in both analyzed subgroups, which are primary and recurrent EOC. These findings may be influenced by several factors, including variability in study design, sample size, treatment regimens, follow-up duration, concurrent medications or interventions, patient demographic characteristics, as well as molecular, genetic, and biomarker heterogeneity of the tumors themselves. EOC exhibits marked intra-tumoral heterogeneity, encompassing both genetic and epigenetic alterations. This biological complexity may result in variable treatment responses and disease progression trajectories, thereby contributing to the observed heterogeneity across study outcomes [40, 41].

Intervention using high temperature in HIPEC is known to be related to the body’s immune system where hyperthermic conditions are able to stimulate natural and adaptive immune responses through the activation of immune modulators such as heat shock proteins [25]. A similar comparative study by Mendivil et al. [23] also found a decreased risk of disease progression [HR 2.1028,95% CI 1.2941–3.4167; *p* = 0.0027] with a comparison of PFS duration in the intervention and control groups of 25.1 months and 20 months, respectively [23]. What needs to be considered based on the forest plot study obtained is the effect of HIPEC on PFS values in patients with recurrent EOC which is not significant and more towards the control group. This may be triggered by study limitations and the diversity of study types used. The overall analysis of these various studies led to the conclusion that HIPEC intervention after CRS was able to extend the duration of PFS in patients with EOC, especially in the primary cancer group.

The safety of using HIPEC in EOC was reviewed based on the AEs that occurred and the OR obtained from the accumulated forest plot. Overall, only 5 of the 15 studies analyzed discussed AEs that occurred post-intervention in experimental and control patients [22, 24, 25, 27, 32]. AEs included in the meta-analysis were leukopenia, neutropenia, nausea, and electrolyte disturbances. The occurrence of nausea was found and discussed in all five studies with the results of the occurrence of nausea at 471/805 and 232/498. The electrolyte disturbances occurred with a total of 513/576 and 210/449 [22, 24, 25, 27]. He (2021), Lei (2020), and Lim (2022) discussed leukopenia with numbers of 340/638 and 136/327, respectively [22, 25, 27]. Finally, for neutropenia, it was found to be 312/517 and 127/251 in the reports [22, 25]. The numbers listed above represent the number of patients/total accumulation of patients in the experimental and control groups respectively.

Hematological complication is a common AEs that can occur in patients undergoing HIPEC treatment. A study conducted by Pintado et al. in 2023 found that 77.1% of patients developed hematological complications, with 8.3% of 96 patients developed leukopenia. Although it is a common AEs, hematological is generally not severe and resolves spontaneously without affecting patients’ mortality or hospital stay [42]. Leukopenia occurrence in patients treated with HIPEC was linked with the use of cisplatin and the combination of cisplatin with paclitaxel [43]. Electrolyte imbalance is a notable complication that can occur post-operatively. A study conducted by Somashekhar et al. in 2022 found that electrolyte imbalance occurs in 16.4% of 1470 patients receiving HIPEC treatment [44]. Electrolyte imbalance complication severity can vary among patients and can be managed with intensive monitoring and correcting electrolyte imbalance. Further research is needed to find the optimal and safe dose to prevent leukopenia complications, protocols regarding managing electrolyte imbalance are also required. Nausea is frequently reported in patients undergoing HIPEC treatment. A study conducted by Argenta et al. in 2013 found that 60% of patients who have nausea complications were linked to carboplatin toxicity. Despite the high incidence number of nausea, it is generally considered tolerable and manageable with medication. It also does not significantly interfere with the feasibility of HIPEC treatment [45].

The final result found that, by assessing the OR, it was found to be more significant in the control group. This implies that fewer AEs occurred in the control group compared to the experimental group with HIPEC. However, it should be emphasized that the AEs were minimal and did not constitute a serious or emergency condition. In addition to effectiveness and safety, another aspect that needs to be considered in the application of HIPEC therapy for EOC patients is its cost-effectiveness value compared to standard therapy. Research by Ji Hyun et al. analyzed the cost-effectiveness of implementing HIPEC in a study setting in Korea [9]. Compared with interval CRS, the use of HIPEC at stage III–IV resulted in an additional quality-adjusted life year (QALY) of 3.64 and an incremental cost of 2600 USD. Total HIPEC resulted in an incremental cost-effectiveness ratio (ICER) of 708.3 USD/QALY, well below the willingness-to-pay (WTP) threshold of the Korean population of 30,496 USD/QALY.

This cost-effectiveness value can increase if applied to patients with less controlled EOC. Other studies by Lim et al. in the USA and Koole et al. in the Netherlands showed that the application of HIPEC in patients with grade III ovarian cancer can save costs by reducing the incidence of PFS, OS, and recurrence as in Table 5 [8, 10]. In the results presented by Kim et al., ICER which is around 708.3 USD/QALY is below the gross domestic product (GDP) per capita of the Indonesian population which is around 3113 USD, and WTP per QALY of the Indonesian population which ranges from USD 2366 to 13,125, indicating its high potential to be a cost-effective therapeutic modality [9, 46, 47]. Studies discussing the feasibility of HIPEC in low- and middle-income countries (LMICs) are still limited. No studies were identified that evaluated the cost-effectiveness of CRS + HIPEC or HIPEC only in the treatment of EOC in LMICs specifically. Nevertheless, we can conclude that countries whose GDP and WTP are lower than these countries find it difficult to apply CRS + HIPEC as a modality in EOC therapy [48]. However, until now, there have been no studies comparing the cost-effectiveness of CRS + HIPEC therapy with economic conditions or standard management of EOC in Indonesia also. Therefore, further tool development and research are needed to identify and optimize the cost-effectiveness and financing regulations in EOC patients for HIPEC therapy in LMICs including Indonesia.

This meta-analysis summarized analyses from RCT studies and observational analytic studies (cohort and case–control), and sensitivity analysis was used to test for heterogeneity. In addition, publication analysis by funnel plot and quantitative analysis of adverse events were performed. The variability of countries, research centers, and various patient characteristics in these studies suggests broad generalizability. Based on the systematic search, this study is the first to describe how HIPEC affects the survival time indicators as well as AEs in patients with EOC, namely OS and PFS with diverse studies. Previous studies only assessed HIPEC in general without specification of NACT without assessment of AEs, as well as other study characteristics as listed in Table 3.

However, there were several limitations in this review. Firstly, there was too much variation in HIPEC treatments including length of treatment (60–90 min), differences in mean temperature (40–43 °C), specific mechanisms (e.g., open/close methods, chemotherapy agents including cisplatin, paclitaxel, mitomycin C), and participants’ sociodemographic characteristics (group of age, races). This inhomogeneity makes it difficult to make direct comparisons between studies and limits the generalization of findings. These differences can also affect the toxicity and effectiveness of therapy, requiring standardization in future research. Secondly, patient comorbidities were not included due to underreporting. Third, there were several studies that reversed the hazard ratio reporting.

Study results have shown that HIPEC on primary EOCs exhibited promising impacts on improved OS and PFS. However, in the case of recurrent EOCs, the efficacy of HIPEC still showed variability, with some studies reporting limited or statistically insignificant benefits. This suggests that HIPEC applicability to recurrent EOCs requires stricter patient selection as well as further evaluation through high-quality studies.

## Conclusion

HIPEC is a novel postoperative cancer therapy that involves intraperitoneal administration of high-temperature chemotherapy to improve OS duration and PFS in patients with EOC. This therapy has shown good results, especially in cancers that have not recurred. No serious AEs were found in HIPEC patients, but minimal AEs such as nausea, neutropenia, leukopenia, and electrolyte imbalance were still quite high after HIPEC administration. Nevertheless, the potential and efficacy of HIPEC in improving OS and PFS should be considered in making it a promising therapy to treat patients with primary and recurrent AE. The application of HIPEC as a modality of chemotherapy therapy in EOCs is also potentially *cost-effective*, especially when applied to EOC patients with higher stages.

As a suggestion, further studies are needed with a wider number of participants and coverage of research areas, especially in the Asian region so that the results obtained are more homogeneous, accurate, and significant. There is also a need for specific studies on elderly cancer patients, acceptance and satisfaction of HIPEC users from both patients and health workers, and regulation of the use of HIPEC as a modality of EOC therapy in women to cancer treatment guidelines in Indonesia.

## Data Availability

Data available within the article. The authors confirm that the data supporting the findings of this study are available within the article.
